# Actions speak louder than words; pediatricians, gynecologists, nurses, and other mothers’ perspectives on the human papillomavirus vaccine: an Istanbul multicenter study

**DOI:** 10.3389/fpubh.2024.1361509

**Published:** 2024-05-02

**Authors:** Burcu Parlak, Funda Güngör Uğurlucan, Emine Gülbin Gökçay

**Affiliations:** ^1^Institute of Health Sciences, Istanbul University, Istanbul, Türkiye; ^2^Department of Obstetrics and Gynecology, Istanbul Faculty of Medicine, Istanbul University, Istanbul, Türkiye; ^3^Department of Social Pediatrics, Institute of Child Health, Istanbul University, Istanbul, Türkiye

**Keywords:** HPV vaccine, physician mothers, attitude, questionnaire, cervical cancer, cervical screening

## Abstract

**Introduction:**

Gynecologists and pediatricians have an essential duty to prevent cervical cancer. In this study, we compared the compliance of gynecologists (*n* = 22) and pediatricians (*n* = 49) with nurse/midwife (*n* = 66) and non-medical moms (*n* = 120) with regards to cervical cancer precautions.

**Methods:**

A questionnaire was used to gather data on their demographics, personal vaccination and screening practices, children’s immunization status, and awareness of cervical cancer prevention.

**Results:**

The findings demonstrated that gynecologists and pediatricians were better than others at understanding the risk factors and prevention of cervical cancer. It was noted that compared to other groups, physician mothers and their offspring had higher vaccination rates (*n* = 13, 18.3%; *n* = 10, 29.4%, respectively). Medical professionals typically provided thorough and accurate answers to informational questions. More frequent Pap smear tests were performed by gynecologists. It was noted that mothers who worked as pediatricians and nurses/midwives neglected their own screening needs.

**Discussion:**

This questionnaire survey sought to ascertain Istanbul’s health professionals’ present opinions regarding HPV vaccination. Healthcare professionals should be the first to receive information on HPV vaccination and cervical cancer incidence reduction. The public could then readily use them as an example.

## Introduction

The most prevalent sexually transmitted infection is called human papillomavirus (HPV) (1). Cervical cancer, the fourth most common cancer in women, is caused by it (2). Every year, about 310,000 women pass away from this; 90% of these deaths take place in developing nations (2). Every year in our nation, there are 1,245 fatal cases of cervical cancer and 2,532 new cases (3). Following its expedited FDA approval in June 2006, the European Medicines Agency authorized Gardasil™ for marketing in the entire European Union in September of that same year (4). The World Health Organization (WHO) lists cervical cancer as a public health issue that needs to be eradicated. To that end, interventions like the evidence-based HPV vaccine and ongoing cervical cancer screening should be implemented widely (2).

Mothers are more prone to cervical cancer awareness. Gynecologists/pediatricians and nurses/midwives are in charge of organizing and carrying out cervical screenings as well as recommending and delivering the HPV vaccination. These reasons led us to focus our study on gynecologist/pediatrician and nurse/midwife mothers. Our H_1_ hypothesis is “Mothers who are pediatricians and gynecologists tend to have their children vaccinated against HPV more than the general population.” In order to increase awareness about cervical cancer that can be prevented by vaccination, we set out to assess mothers’ attitudes and level of knowledge. In previous studies, surveys about HPV vaccines have been conducted with parents, students, nurses, midwives, and doctors. In Turkey, mothers are typically the ones who look after the children. The physician groups that provide information to mothers on this subject are generally pediatricians and gynecologists. In our literature review, we were unable to notice any study comparing the knowledge and attitudes of pediatrician and gynecologist mothers with nurses, midwives, and non-healthcare mothers. Individuals were more likely to get vaccinated or vaccinate their children if they received a favorable recommendation from the their physician. Our goal was to determine whether mothers who work in medicine vaccinated their kids to a degree that would serve as a model for the community. The fact that nurses are more numerous, see patients more frequently, and spend more face-to-face time with them puts them in a great position to provide information and set an example about the HPV vaccine. For this reason, nursing education is also crucial.

Although HPV vaccines are approved by the Ministry of Health in Turkey, national immunization schedule does not include HPV vaccinations, yet. There is a precedent court decision regarding repayment by the Social Security Organization.

## Materials and methods

With permission from the Istanbul Faculty of Medicine Clinical Research Ethics Committee, dated 04/01/2021–1,065, a preliminary study was carried out. The Istanbul Faculty of Medicine Clinical Research Ethics Committee decision, dated 25.02.2022 and numbered 770,003, granted approval for the multi-center study. It is a doctoral thesis.

The authors consulted previous research while creating the questionnaire. The references used to prepare the questionnaire are mentioned on Supplementary File S1–S3.

There were fifteen questions about personal data in the first section of the questionnaire (Supplementary File S1). Twenty-five knowledge questions about HPV, HPV vaccination, risk factors for cervical cancer, and cervical screening tests were included in the second section of the questionnaire (Supplementary File S2). Eight attitude questions about the HPV vaccine and cervical screening were included in the third section of the questionnaire (Supplementary File S3).

In this study, we compared the knowledge and attitudes of pediatricians (*n* = 49) and obstetrician-gynecologists (*n* = 22) about HPV and HPV vaccines with nurse/midwives (*n* = 66) and non-medical mothers (*n* = 120). We requested responses to a three-part questionnaire from gynecologists/pediatricians, nurses, and midwives mothers at all seniority levels who worked at the Istanbul Faculty of Medicine’s Department of Child Health and Diseases, Department of Gynecology and Obstetrics, and Marmara University Pendik Training and Research Hospital between February 2021 and May 2022. Mothers received the majority of the printed surveys, a small portion was sent via social media accounts in the preliminary study.

Mothers who did not work in healthcare were given questionnaires to fill out while in line at the Istanbul Faculty of Medicine’s social pediatrics outpatient clinic, general pediatrics outpatient clinic, and gynecology outpatient clinic. The questionnaires were collected in the same order as the mothers received them.

The survey was distributed without any rewards or punishments for taking part. They agreed to take part voluntarily. Informed consent was added to the survey’s introduction to ensure respondents’ anonymity and their freedom to leave the study at any time. The study contained no identifiers or personal information. They were given rights assurances and had an opportunity to ask questions prior to the interview.

## Statistical analysis

Data analysis was evaluated with the IBM Statistical Package for Social Sciences (SPSS) 15.0 for Windows statistical package program. Nominal (discrete) variables were evaluated with the chi-square test with Yates correction and the Fisher exact probability test. The significance limit was taken as *p* < 0.05 and two-sided. Continuous variables are given as median, standard deviation, minimum, maximum by *t* test and One-way ANOVA, and discrete variables are given as frequency and percentage. The relationship between categorical variables is given as the Phi coefficient or Cramer’s υ coefficient. Statistical calculations were made on a question-by-question basis, calculated only on those who answered that question.

One hundred thirteen mothers received the survey in the pilot study. Ten minutes was found to be the average survey response time. Research issue was “Mothers who are pediatricians and gynecologists tend to have their children vaccinated against HPV more than the general public.” When type 1 error is 5% (bidirectional), and type 2 error is 5% (power 95%), two-way Zα/₂ constant value is 1.96, and the constant value of Zᵦ is 1.645. Those who wanted to vaccinate their children were 56% in the first group and 19% in the second group. The number of samples was calculated as 114 people in total, with 38 people in each group. The equation was:


$$n=\frac{{\left(Z\alpha {/}_{2}+\;Z\beta \right)}^{2}[(P_{1}\left(1-P_{1}\right)+P_{2}\left(1-P_{2}\right)}{{\left(P_{1}-P_{2}\right)}^{2}}$$

$$n=\frac{{\left(1.96+1.645\right)}^{2}[(0.56\left(1-0.56\right)+0.19\left(1-0.19\right)}{{\left(0.56-0.19\right)}^{2}}$$

n=38

Mothers whose daughters were born after 1980 and whose sons were born after 1983 participated in the study. These mothers were gynecologists, obstetricians, pediatricians, nurses, midwives and non-healthcare workers. The chosen ones were mothers whose daughters were no older than 26 in 2006, the year the vaccine was first approved. In other words, mothers who had daughters were born in as early as 1980 (2006–26 =) were included. The birth date of 1983 (2009–26 =) was used as the basis for boys since Gardasil™ was authorized for use in 2009 for boys aged 9 to 26.

Repeated surveys, inconsistent responses, fathers, physicians other than obstetrics and pediatrics or mothers without children, and those who answered only demographic questions were not included in the study.

## Results

All in all, 276 surveys were completed, and 257 of them were evaluated, 19 were excluded. Group 1 consisted of gynecologists (*n* = 22) and pediatricians (*n* = 49). Group 2 included mothers who were not healthcare professionals (*n* = 120). Mothers who were nurses or midwives made up Group 3 (*n* = 66).

### Demographic characteristics

Marital status, mean age, average child age, number of children over 9 years of age, smoking, and presence of cervical cancer in the immediate circle were found to be similar in all groups. When the sociodemographic characteristics of the groups were compared, no difference was found except of active working. Compared to Group 2, Groups 1 and 3 had higher rates of active work (97.2, 98.5, 44.2% respectively, p = 0.0001). 144 mothers with children age appropriate for HPV vaccination (over 9 years old) participated in our study. Demographic characteristics of the groups are presented in Table 1.

**Table 1:** Demographic characteristics of the groups.

		Group 1 (*n* = 71) (%100)	Group 2 (*n* = 120) (%100)	Group 3 (*n* = 66) (%100)	Mean	*p*
Marital status	Single *n* (%)	4 (5.6)	5 (4.2)	3 (4.6)		
	Married *n* (%)	63 (88.8)	114 (95)	60 (90.9)		0.375*
	Divorced *n* (%)	4 (5.6)	1 (0.8)	3 (4.5)		
Age yrs. mean (SD) (min–max)		41.69 (8.5) (28–65)	40.62 (8.3) (23–68)	39.05 (7.4) (24–59)	40.51 (8.2) (23–68)	0.162**
Children age yrs. mean (SD) (*n*)		11.7 (8.67) (115)	13.4 (11.8) (244)	9.8 (9.8) (105)	12.2 (9.3) (464)	0.243**
Anyone with a history of cervical cancer in your close circle *n* (%)		10 (14)	14 (13)	6 (9.5)		0.709*
Smoking	No	58 (81.7)	83 (69.2)	46 (69.7)		
	Yes	6 (8.5)	25 (20.8)	9 (13.6)		0.053*
	Occasionally	6 (8.5)	10 (8.3)	11 (16.7)		
Those with children over 9 years old *n* (%)		34 (47.9)	75 (62.5)	35 (53)		0.132*
Active work *n* (%)	Yes	69 (97.2)	53 (44.2)	65 (98.5)		0.0001*
	No	2 (2.8)	59 (49.2)	1 (1.5)		

*Pearson Chi-square test. **One-way ANOVA.

Group 1 generally have over 10 years of professional experience. 18 participants (25.4%) of Group 1 have 10 years or less of professional experience, 34 (47.9%) participants of Group 1 have 10–20 years of professional experience, and 17 (23.9%) participants of Group 1 have more than 20 years of professional experience.

It was determined that Group 1 mostly worked in university/education and research hospitals (*n* = 39; 54.9%), followed by state hospitals (*n* = 13; 18.3%). Others work in private universities, private hospitals, private clinics and private offices (*n* = 19; 26.8%).

### The answers about attitude

Group 1 was more likely to receive the HPV vaccine (*n* = 13, 18.3%). Group 1 is different from the others, significantly. HPV vaccination status of Group 2 (*n* = 5, 4.2%) and Group 3 (*n* = 1, 1.5%) is similar in pairwise comparison (*p* = 0.421 Fisher’s Exact test) (Table 2). Although physician mothers with 10–20 years of professional experience received HPV vaccination more often (10.3%) than other physicians, no statistical difference was found. According to the Phi-coefficient, an association of approximately 18% was found between the family’s monthly income and HPV vaccination status (Fisher’s exact test, *p* = 0.009). When monthly income was compared with the reasons for not getting the vaccine, as income increased, the number of people citing price as a justification decreased.

**Table 2:** Have you had the HPV vaccine?

	Group 1 (*n* = 71)	Group 2 (*n* = 120)	Group 3 (*n* = 66)	*N* (%)	*p* = 0.0001
Yes *n* (%)	**13 (18.3)**	5 (4.2)	1 (1.5)	19 (7.4)	
No *n* (%)	58 (81.7)	112 (93.3)	65 (98.5)	235 (91.4)	
No answer *n* (%)	0	3 (2.5)	0	3 (1.2)	
*N* (%)	71 (100)	120 (100)	66 (100)	257 (100)	

Pearson Chi-square test, χ2 = 17.164, SD = 2.

Group 1 is different from the others; physician mothers give more attention to their children. Their children had been vaccinated at a high rate (*n* = 10, 29.4%). Groups 2 (*n* = 1, 1.4%) and 3 (*n* = 0) whose children are eligible for vaccination have similar attitudes about vaccinating their children against HPV. (*p* = 1,000 Fisher’s Exact test) (Table 3).

**Table 3:** Mothers who had their children vaccinated against HPV among those whose children are suitable for vaccination.

	Group 1 (*n* = 34,%100)	Group 2 (*n* = 75,%100)	Group 3 (*n* = 35,%100)	*N* (%)	*p* = 0.0001
Yes *n* (%)	10 (29.4)	1 (1.4)	0 (0)	11 (7.6)	
No *n* (%)	24 (70.6)	74 (98.6)	35 (100)	133	
No answer	0	0	0	0/144	

Pearson Chi-square test, χ2 = 22.136, SD = 2.

Group 1 with over 10 years of professional experience had over 9 years old children. It was found that physicians with more than 20 years of experience had their children vaccinated against HPV at a higher rate (21 years and above: *n* = 8/16, (%50), *p* = 0.037) (Table 4). In Group 1, among those with children aged 9 and over, the rates of their children’s HPV vaccination rates analyzed based on their place of employment. The rate of having their children vaccinated against HPV was higher in public hospitals (*n* = 5; 62.5%), and the lowest rate was among those working in university hospitals (*n* = 1; 7.7%) Public and University Hospital are different pairwise comparisons (Fisher’s Exact test, *p* = 0.014) (Table 5). According to the Phi-coefficient, an association of approximately 32% was found between the monthly income of the family and the status of having their child vaccinated against HPV (Fisher’s exact test, *p* = 0.001).

**Table 4:** Based on the professional experience of Group 1, have you vaccinated your children aged 9 and above with the HPV vaccine?

	No	Yes	*N* (%)	*p* = 0.037
11–15 years *n* (%)	5 (100)	0	5	
16–20 years *n* (%)	11 (84.6)	2 (15.4)	13 (100)	
21 years and above *n* (%)	8 (50)	8 (50)	16 (100)	

Pearson Chi-square test, χ2 = 6.582, SD = 2.

**Table 5:** Answers of the question “Have you had your child vaccinated against HPV?” according to place of employment among those with children aged 9 and over in Group 1.

Working place	No *n* (%)	Yes *n* (%)	*N* (%)	*p* = 0.028
Public Hospital *n* (%)	3 (37.5)	5 (62.5)	8 (100)	
Private Hospital/Private Clinic/Private Practice/Private University Hospital *n* (%)	9 (69.2)	4 (30.8)	13 (100)	
University/Education Research Hospital *n* (%)	12 (92.3)	1 (7.7)	13 (100)	

Pearson Chi-square test χ2 = 7.184, SD = 2.

Group 1 wishes to vaccinate their children against HPV at a rate that is significantly higher than the others (*n* = 47, 66.2%). Groups 2 (*n* = 32, 26.7%) and 3 (*n* = 32, 48.5%) are similar in terms of wanting to have their children vaccinated against HPV (Table 6). Group 1 wants to vaccinate their children more than the others (*n* = 49, 70.1%), which is substantially different. When asked how many of their children they would vaccinate, Group 2 (*n* = 35, 29.2%) and Group 3 (*n* = 29, 43.9%) gave comparable responses (*p* = 0.372 Fisher’s Exact Test in pairwise comparisons) (Table 7). 28 (57%) wanted their daughters, 12 (24.5%) wanted their sons, and 9 (18.4%) wanted both their sons and daughters vaccinated.

**Table 6:** Do you want your child to get the HPV vaccine?

	Group 1 (*n* = 71)	Group 2 (*n* = 120)	Group 3 (*n* = 66)	*N* (%)	*p* = 0.0001
No *n* (%)	16 (22.5)	52 (43.3)	28 (42.4)	96 (37.4)	
Yes *n* (%)	47 (66.2)	32 (26.7)	32 (48.5)	111 (43.2)	
Do not know *n* (%)	0 (0)	2 (1.6)	0 (0)	2 (0.8)	
No answer *n* (%)	8 (11.3)	34 (28.3)	6 (9.1)	48 (18.7)	
*N* (%)	71 (100)	120 (100)	66 (100)	257	

Pearson Chi-square test, χ2 = 19.297, SD = 2.

**Table 7:** How many of your children do you vaccinate?

	Group 1 *n* (%)	Group 2 *n* (%)	Group 3 *n* (%)	*N* (%)	*p* = 0.004
None of them	16 (22.5)	37 (30.8)	29 (43.9)	82 (31.9)	
One of them	30 (42.3)	15 (12.5)	15 (22.7)	60 (23.4)	
All of them	19 (27.8)	20 (16.7)	14 (21.2)	53 (20.6)	
No answer	6 (8.5)	48 (40)	8 (12.1)	62 (24.1)	
*N* (%)	71	120	66	257	

Pearson Chi-square test χ2 = 15.135, SD = 4.

The groups were found to be similar in terms of the distribution of the brands (Cervarix™, Gardasil™) of the vaccines and the recommended doses (*p* = 0.075, *p* = 0.1, respectively).

Among all groups, 55 (21%) participants stated that they did not receive the HPV vaccine because it was unnecessary. 50 (18.2%) of them had not vaccinated because of the price. 29 (10.5%) participants had not received the HPV vaccine due to side effects. The most common reasons for not getting the vaccine in the groups were 28 (42.4%) in Group 3 because it was expensive, 27 (22.5%) in Group 2 because they found the vaccine unnecessary, and 17 (23.9%) in Group 1 because the person is older (Table 8).

**Table 8:** If you have not had the HPV vaccine, what is the reason?

	Group 1 *n* (%)	Group 2 *n* (%)	Group 3 *n* (%)	*N* (%)
Allergy	0	1 (0.8)	0	1
Do not know	0	18 (15)	1 (1.5)	19 (7.4)
Price	10 (14.1)	12 (10)	**28 (42.4)**	**50 (18.2)**
Unnecessary	12 (16.9)	**27 (22.5)**	16 (24.2)	**55 (21)**
Neglect	9 (12.7)	1 (0.8)	4 (6.1)	14 (5.5)
Side effects	4 (5.6)	21 (17.5)	4 (6.1)	**29 (10.5)**
Age	**17 (23.9)**	2 (1.6)	7 (10.6)	25 (9.7)
No answer	19 (26.8)	38 (31.7)	6 (9.1)	63 (24.5)
N (%)	71 (100)	120 (100)	66 (100)	257

Group 2 recommends significantly less HPV vaccine to their close circle (*n* = 46, 38.3%) than the others. Despite the similarities between Groups 1 and 3, Group 1 advises the HPV vaccine to their circle at a higher rate (*n* = 60, 84.5%) (Table 9).

**Table 9:** Do you recommend the HPV vaccine to your patients or your close circle?

	Group 1 *n* (%)	Group 2 *n* (%)	Group 3 *n* (%)	*N* (%)	*p* = 0.0001
Yes	60 (84.5)	46 (38.3)	41 (62)	147 (57.2)	
No	11 (15.5)	44 (36.7)	18 (27.3)	73 (28.4)	
No answer	0	30 (25)	7 (10.6)	30 (11.7)	
N (%)	71	120	66	257	

Pearson Chi-square test, χ2 = 20.544, SD = 2.

The groups are similar in terms of having regular Pap smear tests (*p* = 0.167). It was discovered that gynecologists (*n* = 17, 77.3%) paid considerably more attention to have routine Pap smear tests than pediatricians (*n* = 20, 41.7%) (Table 10). Pap smear test rates were found to be similar in all groups (Table 11). The Phi-coefficient showed a 15.2% correlation between routine Pap-smear testing and knowledge that HPV causes cervical cancer (Fisher’s exact test, *p* = 0.035).

**Table 10:** The rate of regular Pap smear tests regarding of branch in Group 1.

	Pediatrist *n* (%)	Gynecologist *n* (%)	*p* = 0.012
No	28 (58.3)	5 (22.7)	
Yes	20 (41.7)	17 (77.3)	
Not answering	1 (2)	0	
*N* (%)	49	22	

Yates’ Chi-square (continuity correction) test: χ2 = 6.313, SD = 1.

**Table 11:** The rate of regular Pap smear tests in each group.

21–65 years old range	Group 1 *n* (%)	Group 2 *n* (%)	Group 3 *n* (%)	*N* (%)	*p* = 0.167
No	33 (46.5)	61 (50.8)	34 (51.5)	128 (49.8)	
Yes	37 (52.1)	39 (32.5)	30 (45.5)	106 (41.3)	
No answer	1 (1.4)		2 (3)		
*N* (%)	71	120	66	257	

Pearson Chi-square test, χ2 = 3.587, SD = 2.

When univariate analyzes were performed, the results of the statistical tests were found as; age (*p* < 0.0001), marital status (*p* = 0.023), professional experience (*p* < 0.0001), smoking (*p* = 0.519), active employment (*p* = 0.195), number of children (*p* = 0.134). 11 participants out of 257 did not answer the questionnaire. Of the remaining 246 people, 235 said “no” and 11 said “yes” to having their children vaccinated. Since children over the age of 9 were vaccinated, 113 of the 257 people were excluded because they had children under the age of 9, and univariate analysis then multivariate logistic regression analysis were performed on the remaining 144 participants. Of these 144 people, 127 “did not vaccinate their children” (92%), and 11 of them “had their children vaccinated “(8%).

In this case, univariate analyzes results were found as; age (*p* = 0.004), marital status (*p* = 0.02), title (*p* = 0.665), monthly income (*p* = 0.013), and professional experience (*p* = 0.037).

Multivariate logistic regression was performed on variables with *p* < 0.05. The dependent variable was “vaccinated/did not vaccinate her child”. Independent variables were age, marital status, workplace, monthly income, and professional experience which were found as statistically significant in the univariate analysis.

According to the logistic regression analysis; Classification Table: 70.6%, Nagelkerke R = 0.284, Omnibus Test of Model *p* = 0.006, Hosmer Lemeshow Test *p* = 0.69 were found. The only significant variable for the situation of “vaccinating your child” was “professional experience” (*p* = 0.024). Exp (ᵦ) = 0.65 (Risk). 95% CI for Exp (ᵦ) (1.258, 33.596). When the professional experience increases, the tendences for vaccinating the child also increases. On the other hand, one person who has her child vaccinated is a housewife, the other 2 doctors are people with 16–20 years of professional experience, and 8 doctors are people with 21 years or more of professional experience.

### The answers about information

The other books and broadcastings (TV, newspaper, magazine, non-scientific journal) (*n* = 54, 21%) was at the top of all sources about HPV in all groups. “Social media” was the first source of reference in Group 2 (*n* = 43, 35.8%). Medical school was the most frequently mentioned source (*n* = 25, 35.2%) in Group 1. In Group 3, the most common answer regarding the source was “the other books and broadcastings” (*n* = 28, 42.4%) (Supplementary File S4).

All groups were different from each other in terms of the response to the query regarding the age range of the HPV vaccine target population. Group 1 gave the most correct answers (*n* = 66, 93%). Group 3 was in second place (*n* = 43, 68.3%), while Group 2 answered the least (*n* = 54, 45%) correct response rate (Supplementary File S4). Every group has different information regarding the cost of the HPV vaccine. While Group 3 stated that it was not reasonable at a higher rate (*n* = 44, 66.7%), Group 1 stated that it was reasonable at a higher rate (*n* = 15, 21.1%) (Supplementary File S4). Group 2 were less aware that multiple sexual partners increased the risk of HPV (Fisher’s Exact Test *p* = 0.011, Group 1 and 2 in pairwise comparison) (Supplementary File S4). Group 1 knew 100% correctly that HPV was sexually transmitted. Pairwise comparisons revealed that while Groups 1 and 2 were different (Fisher’s Exact test, *p* = 0.005), the other groups were similar (Supplementary File S4). The difference between Groups 2 and 3 was found to be significant regarding whether using a condom reduces the risk of HPV. Group 3 (*n* = 55, 83.3%) was more aware that condoms reduce the risk of HPV than Group 1 (*n* = 56, 79%) (Supplementary File S4). Group 2 (*n* = 84, 70%) knew that HPV could cause cervical cancer compared to the other groups at a lower rate. Group 1 and Group 3 responded similarly (Supplementary File S4). Compared to the other groups, Group 1 (*n* = 70, 98.6%) was more aware that an individual could have HPV infection and go years without realizing it (Supplementary File S4). Group 1 (*n* = 69, 97.2%) had the most knowledge that HPV was a common infection, while Group 3 (*n* = 50, 75.8%) had the least awareness of this fact (Supplementary File S4). Group 2 had the lowest rate of knowledge (*n* = 77, 64.2%), whereas Group 1 knew the most (*n* = 70, 98.6%) about the variety of HPV types (Supplementary File S4). Group 1 (*n* = 69; 97.2%) knew more than the other groups that sexual intercourse at an early age increases the risk of HPV. Group 2 had the lowest knowledge on this subject (*n* = 62; 51.7%) (Supplementary File S4). Compared to the other groups, Group 1 was considerably (*n* = 63, 88.7%) more aware that HPV could not yet be treated with antibiotics or antivirals. This rate was lowest in Group 3 (*n* = 26; 39.4%) (Supplementary File S4). Group 1 knew that HPV also infects men at a higher rate than the other groups (*n* = 67, 94.4%). Group 2 knew this issue the least (*n* = 70; 58.3%) (Supplementary File S4). Group 1 knew (*n* = 71, 100%) that the symptoms of HPV were not always visible. Groups 2 and 3 responded at similar rates (*n* = 82, 68.3%; *n* = 51, 77.3%, respectively) (Supplementary File S4). More people did not know that HPV causes genital warts in Group 2 than the other groups (*n* = 12, 10%). Group 1 and Group 3 were found to be similar (Supplementary File S4). Although Group 1 answered that HPV usually heals without treatment more correctly than the other groups, the majority of them answered wrong (*n* = 27, 38%) Groups 2 and 3 responded at similar rates (*n* = 6, 5%; *n* = 5, 7.8%, respectively) (Supplementary File S4). Group 1 knew that vaccinated girls should continue to have Pap smear test regularly correctly (*n* = 71, 100%). The answers of Groups 1 and 2 (*n* = 88, 73.3%) were significantly different. Groups 1 and 3 or Groups 2 and 3 are similar between themselves (Supplementary File S4). All groups know at similar rates that the HPV vaccine protects against many types of cervical cancer (Supplementary File S4). Group 3 had a higher rate of incorrect answers to the statement that someone who has been vaccinated against HPV will never develop cervical cancer (*n* = 15, 22.7%). There was a difference between Groups 1 and 3 (χ2 = 4.121, sd = 1, *p* = 0.042 Yates’ Chi-square). Groups 2 and 3, Groups 1 and 2 gave similar responses among themselves (Supplementary File S4). Group 2 had a significantly higher rate of ignorance (*n* = 36, 30%) regarding the possibility that HPV could also cause other types of cancer (Supplementary File S4). Twenty-six (21.7%) individuals in Group 2 were unaware that the HPV vaccine offers protection against vaginal warts. Group 3 gave the least incorrect answers (*n* = 7, 10.6%) (Supplementary File S4). Group 1 (*n* = 64, 90.1%) was significantly more likely to know that men/boys should also be vaccinated compared to Group 3 (*n* = 41, 62.1%). Group 2 answered similar to other groups (Supplementary File S4). At least Group 2 knew that HPV vaccine is administered in 2 doses, 6 months apart, between the ages of 9–14 (*n* = 53, 44.2%). Other groups are similar between themselves (Supplementary File S4). Group 1 (*n* = 57, 80.3%) knew the most about the HPV vaccine, which is administered to individuals 15 years of age and older in three doses at 0, 2, and 6 months. Group 2 was at least aware (*n* = 52, 43.3%) Groups 1 and 2 answered differently (Supplementary File S4).

As observed in Additional File 4, the prevalence of missing answers in Group 2 is quite high compared to the other groups due to the lack of knowledge about HPV and the HPV vaccine among those who do not work in the healthcare industry. For this reason, most of them wrote “I do not know” next to the questions. Since HPV is popularly known as the wart virus or cervical cancer virus, it was thought that the definition of “HPV” increased the number of unanswered questions in face-to-face surveys.

## Discussion

We analyzed survey data about knowledge of HPV and attitudes toward the HPV vaccine and cervical screening test from 71 mothers who were physicians, 66 mothers who were nurses or midwives, and 120 mothers who were not medical professionals. As anticipated, our findings demonstrated that mothers who work as pediatricians and gynecologists were more successful than mothers in other groups in getting themselves and their kids vaccinated against HPV. Our study included 13 (18.3%) vaccinated physicians. In Turkey, national immunization schedule does not include HPV vaccinations, yet. That’s why vaccination rates are generally low. Although physician mothers had cervical cancer screening tests done more regularly than other groups, unfortunately this difference was not significant and was a low rate (*n* = 37, 52.1%). Lubeya MK et al., conducted a study in Zambia in 2022 with 121 doctors, including 26 (21.5%) gynecologists, 18 (14.9%) pediatricians, and 24 (19.8%) surgeons. Sixty-nine (65.3%) of the physicians in their survey study had more than 10 years of clinical experience. A total of 66 (54.6%) physicians recommended HPV vaccination (5). On the contrary, physicians were more likely to recommend the vaccine in our study. As the number of experienced doctors increases, vaccination recommendations seem to increase. Kurtoğlu E et al. conducted a survey with 53 family physicians in 2013, and it was determined that 17 (32.1%) physicians wanted to get the vaccine for their daughter, and 14 (26.4%) physicians wanted to get the vaccine for their son (1). The rate of physicians recommending vaccination to their patients was found to be only 33 (62.3%). It was observed that 32 (60.4%) of family physicians had insufficient knowledge about HPV vaccine (1). It can be thought that the awareness of pediatricians and gynecologists is higher than the family physicians in this study, as doctors in our study wanted to vaccinate their children at a higher rate and recommended the vaccine to their patients. It is noteworthy that as the rate of physicians updating their knowledge decreases, the rate of vaccine acceptance and recommendation to their patients decreases.

In our study, Group 1 knew that vaccinated girls should continue to have Pap smear test regularly correctly (*n* = 71, 100%). Group 1 is fully aware that HPV causes cervical cancer. Almazrou S, et al. did a study in Arabia in 2020. In this research, 58 (33%) physicians had professional experience more than 10 years. In his study conducted with 121 (70%) pediatricians and 52 (30%) family physicians (6). 102 (59%) physicians knew that vaccinated girls should continue to have Pap smear test regularly correctly. 6 (3.5%) physicians received HPV vaccination. These rates were low comparing with our study. The reason for this low rate may be that the vaccine is not on the national schedule in Arabia. 142 (82%) physicians said to want their daughters to be vaccinated against HPV. This rate was higher than our study. Physicians with over 10 years of experience were more likely to have a higher level of knowledge than those with less experience (6). In our study, we found that the tendency to vaccinate children increases as professional experience increases. These findings also support our study results.

In the survey study conducted by Katsuta T, et al. in 2019 via e-mail with 148 physicians, including 63 pediatricians, and 14 gynecologists, answered the questions. The median experience of physicians was 30 years. 26 (21%) physicians, 11 (22%) pediatricians, and 5 (36%) gynecologists recommend HPV vaccination to adolescents. These rates were lower than our study. Overall, Japanese physicians reported that HPV vaccine recommendations would improve most with policy changes (7). In Group 2 of our study, 5/120 (4.2%) of the mothers received a vaccination. This rate exceeded the vaccination rate of just 11 (1.2%) out of 909 Japanese mothers in the survey study conducted by Suzuki Y et al. It has been reported that one of the main barriers to HPV vaccination in Japan is vaccine hesitancy (8). Social media was the first source in Group 2. Della Polla G, et al. conducted a research with 435 parents, 57.9% of them reported that they had vaccinated their child against HPV and one-third (33.3%) participants were hesitant. Moreover, 56.7% of the remaining intended to vaccinate their child against HPV. In contrast to our results, the most reported source was health-care provider (63.2%), and the second most popular were internet and social media (42.1%) (9). In Italy, the fact that the public learns information from doctors rather than social media and the HPV vaccine is included in the national immunization schedule may explain why vaccination rates are higher than in our study.

In Chen S, et al.’ survey study conducted with 2074 physicians in 2022, 20 surveys were disqualified, 36% gynecologists and 64% healthcare workers were evaluated in terms of HPV vaccine knowledge and recommendation. 68% of the participants stated that they recommended the HPV vaccine (10). This rate was low comparing with our study. The reason for this low rate may be that the vaccine is not on the national schedule in China. They thought that awareness, and knowledge level are lower in Southern China.

We did not find a relationship with vaccination against HPV and a family history of cancer. In the study of Walter LA, et al., there were 6 (7.6%) people with a family history of cervical cancer, and 20 (24.7%) vaccinated participants. Although this rate seems higher than our study, HPV vaccine is included in the national immunization schedule in Alabama (11). Yörük S, et al. conducted a survey in 2016 to examine the knowledge, attitudes and behaviors of female students studying at the faculty of health sciences and medicine regarding HPV, cervical cancer, and HPV vaccine. 92.7% of medical faculty students told that HPV is the causative agent of cervical cancer. 58.2% of nurse/midwife students knew that HPV caused cervical cancer. 6 (0.9%) students were vaccinated. They found that students who had a relative with cervical cancer were more likely to consider getting vaccinated. The reasons for neglecting vaccination were being unaware of vaccine (34.8%), price of vaccine (22.2%), side effects of it (17.4%), and giving up vaccination (15.5%). The HPV knowledge of the medicine students attending the faculty of was higher compared to the other students (12). These results overlap with ours except for the cervical cancer relationship.

In a survey of 704 mothers by Mendes Lobão W et al., 83% mothers had Pap smear test regularly. HPV vaccine acceptance was 92.8% for their daughters and 85.9% for their sons in that study. These were higher rate according to our study. 30% parents knew that HPV vaccine prevents genital warts. This knowledge’s rate is low than ours. The most common reason for not vaccinating a child was found to be not vaccinating at school. HPV vaccine was included in the National Immunization Program in Brazil (13).

Smolarczyk K et al.’ conducted a survey study in Poland in 2021 with 639 doctors, including 31.8% dermatologists, 32.1% gynecologists, 0.2% family physicians, and 33.8% pediatricians. In contrast to our study, 132 (20.7%) physicians, including 47 (23.2%) skin and venereal disease specialists, 51 (24.9%) gynecologists, and 32 (14.8%) pediatricians knew the HPV vaccine target population age. Furthermore, 53 (8.3%) physicians, including 20 (9.9%) dermatologists, 24 (11.7%) gynecologists and 8 (3.7%) pediatricians knew that HPV is transmitted was answered correctly. The dose of the HPV vaccine was known by 266 (41.6%) physicians, including 60 (29.6%) dermatologists, 78 (38%) gynecologists, 121 (56%) pediatricians correctly. 133 (66.5%) dermatologists, 153 (75.4%) gynecologists, and 134 (64.1%) pediatricians recommended the vaccine to their relatives. These rates were also lower than ours (14). Nagase Y, et al. conducted with 293 gynecologists, 248 (84.6%) gynecologists reported that they recommended HPV vaccination to their patients. Gynecologists vaccinated 11 of their 30 daughters (36.7%) against HPV (15). This was slightly better than our result of 10/34 (29.4%), even though it was in Japan when the vaccine was on hold. In their survey study with 318 midwives and nurses in 2021, Ebu NI, et al. found that 176 (55.3%) nurse-midwives had at least one Pap smear test, and 142 (44.7%) participants had no test. 56 (17.6%) participants were vaccinated, and 262 (82.4%) were not vaccinated (16). Although the HPV vaccine is not included in the national immunization schedule in Ghana, the vaccination rate is higher than in our study.

In contrast to our results, at the survey study conducted by Lin Y et al. with nurse students in 2022, 75.4% or nurses stated that HPV was not treated with antibiotics. 70.9% of students knew that vaccinated girls should continue to be screened for cervical cancer. Approximately 2/3 (64.6%) of the students do not know that HPV infection can be asymptomatic (17). Although the rates were determined better in our study, it seems that nurses should receive training about HPV and the HPV vaccine generally.

Karasu et al. (18) researched HPV vaccination attitude and knowledge at 499 nurses. Their vaccination status was 26 (4.3%). 237 (52.8%) nursing students reported that they were considering vaccinating their children. Of them, 86% were aware that HPV is a sexually transmitted infection. 59% of participants in their study knew that HPV infection could be asymptomatic. Of the nurses, 18.6% (*n* = 66) had smear tests performed. Their reasons for not getting vaccinated were: they were not at risk of HPV infection (*n* = 106, 34.9%), some of them said they were unaware of the vaccine (*n* = 83, 26.8%), 23 (7.4%) of the participants said that the vaccine had many side effects, 4 of them (1.3%) answered that the government did not cover the cost of vaccination (18). The fact that pap tests and vaccination rates are as low as ours indicates that nurses require training.

In the survey study conducted by Adesina KT et al. in 2018 with mothers of adolescent daughters in Nigeria, 161 (34.3%) mothers stated that HPV infection was sexually transmitted, 190 (40%). 40.4% mothers knew that it caused cervical cancer, and 162 participants (34.5%) knew that using a condom could prevent transmission. In this study, 1.1% mothers said that boys could also be vaccinated, and 9 (1.9%) participants had their children vaccinated. 211 (44.9%) of them stated that they wanted to vaccinate their children. 45 (9.6%) of them knew that the vaccine could prevent genital warts and 120 (25.5%) mothers knew that it could prevent cervical cancer. They obtained information from doctors (*n* = 80, 28.1%), mass media (*n* = 61, 21.4%), health meetings (*n* = 60, 21.1%), from newspapers and magazines (*n* = 43, 15.1%), from their peers and parents 11 (3.9%), 10 (3.5%), (*n* = 18, 6.3%) from social media, and 2 (0.7%) from their relatives (19). Unfortunately, in our society, social media has been found to be the preferred source of information rather than physicians. Shetty S, et al.’ conducted a survey study with medical students (43.5%) followed by dental (27.9%), nursing (21.1%). Faculities (42.1%) were the most common information source followed by TV/internet (12.1%), family/friends (4.9%), and physician (2.9%). Most students (78%) knew HPV transmission by sexual route. 25.8% students were aware that HPV infection could be asymptomatic. 62.6% students stated that HPV could affect males. Only 37.2% of them were aware that HPV could cause oropharyngeal cancer. 49.5% of students knew that using a condom could prevent HPV infection. 6% of the students had got the HPV vaccine (20). The fact that the vaccine is not in the national immunization schedule in India may be one of the reasons for the low rate of vaccination.

Gynecologists were found to be more attentive in terms of regular smear tests compared to pediatricians. Although physician mothers with 10–20 years of professional experience received HPV vaccination more often than other physicians, no statistical difference was found. In contrast, in the Hershkovitz G et al. study, less experienced physicians were vaccinated more frequently and gynecologists were screened at the same rate as other physicians (21).

This is, as far as we are aware, the first study to look at HPV awareness among nurses, pediatricians, obstetricians/gynecologists, and mothers who are not in the medical field. Regretfully, attitudes regarding cervical cancer prevention have sadly fallen behind the curve for most healthcare professionals.

## Strengths and limitations

There are some limiting aspects of our study. Some participants were reluctant to write their names and phone numbers because they thought it was related to their private lives. The name, telephone and e-mail address sections added after the preliminary study may have caused bias in answering. HPV is colloquially known as the wart virus or cervical cancer virus, the definition of “HPV” was thought to increase the number of unanswered questions in face-to-face surveys. It was observed that there was generally little information about HPV, and it was concluded that awareness would increase if a study was conducted before and after the training. In our study, the education levels of groups were not questioned, but their professions were asked. 2.3 times more participants than the sample calculated in the preliminary study participated in our research. Pediatrician and gynecologist mothers were included in the physician mothers group, the calculated sample size was exceeded, but a study including family physician mothers could also be considered. Having the sample from two esteemed universities—one on the European side and the other on the Anatolian side—in Istanbul, a multicultural city, is one of the research’s advantages. In the initial social media study, the participants’ response rate was higher when they were not required to provide their name, phone number, or email address. The study has social significance for improving vaccination coverage with the help of these experts.

## Conclusion

Based on the data we obtained from the studies we compared, we thought that healthcare professionals did not make enough efforts to prevent cervical cancer. Although physicians recommend the vaccine at a higher rate, they are reluctant to encourage patients to get vaccinated for reasons such as cost concerns. Some physicians regrettably think the vaccine is unnecessary. The reason for the low vaccination frequency in our sample group may be that the importance of vaccination is not yet fully understood among healthcare professionals. The fact that gynecologists and pediatricians are well-versed in the HPV vaccine plays a significant role in their willingness to recommend it to patients and their acceptance of it.

It is necessary to equip physicians in all branches with knowledge who treat patients who may be affected by HPV-related diseases. To encourage behavioral change in young people, opportunities for discussions about sexuality and other culturally sensitive issues should be established with health professionals who possess the requisite knowledge and expertise about cervical cancer. Social media is a valuable resource for information about public health, but it can be challenging to weed out misleading material, so it’s critical that the appropriate regulations and inspections are put in place now.

The importance of matching words and deeds can be taught to medical professionals. Taking action can help to achieve success, to build resilience, and to make a positive impact in the world.

## Data availability statement

The original contributions presented in the study are included in the article/Supplementary material, further inquiries can be directed to the corresponding author.

## Ethics statement

The Istanbul Faculty of Medicine Clinical Research Ethics Committee decision, dated 25.02.2022 and numbered 770003, granted approval for the multi-center study. Written informed consent from the participants was not required to participate in this study in accordance with the national legislation and the institutional requirements.

## Author contributions

BP: Conceptualization, Data curation, Formal analysis, Investigation, Methodology, Resources, Validation, Visualization, Writing – original draft, Writing – review & editing. FU: Conceptualization, Data curation, Formal analysis, Methodology, Resources, Validation, Writing – review & editing, Writing – original draft. EG: Conceptualization, Data curation, Formal analysis, Investigation, Methodology, Resources, Validation, Visualization, Writing – review & editing, Writing – original draft.
