# Corrigendum: Actions speak louder than words; pediatricians, gynecologists, nurses, and other mothers' perspectives on the human papillomavirus vaccine: an Istanbul multicenter study

**DOI:** 10.3389/fpubh.2024.1475093

**Published:** 2024-08-14

**Authors:** Burcu Parlak, Funda Güngör Uğurlucan, Emine Gülbin Gökçay

**Affiliations:** ^1^Institute of Health Sciences, Istanbul University, Istanbul, Türkiye; ^2^Department of Obstetrics and Gynecology, Istanbul Faculty of Medicine, Istanbul University, Istanbul, Türkiye; ^3^Department of Social Pediatrics, Institute of Child Health, Istanbul University, Istanbul, Türkiye

**Keywords:** HPV vaccine, physician mothers, attitude, questionnaire, cervical cancer, cervical screening

In the published article, there was an error in the final author list and affiliations. Instead of

“Burcu Parlak^*^, Funda Güngör Uğurlucan and Emine Gülbin Gökçay

Institute of Health Sciences, Istanbul University, Istanbul, Türkiye,” it should be

“Burcu Parlak^1*^, Funda Güngör Uğurlucan^2^ and Emine Gülbin Gökçay^3^

^1^Institute of Health Sciences, Istanbul University, Istanbul, Türkiye

^2^Department of Obstetrics and Gynecology, Istanbul Faculty of Medicine, Istanbul University, Istanbul, Türkiye

^3^Department of Social Pediatrics, Institute of Child Health, Istanbul University, Istanbul, Türkiye”

The authors apologize for this error and state that this does not change the scientific conclusions of the article in any way. The original article has been updated.

