# Supplementary material for: Actions speak louder than words; pediatricians, gynecologists, nurses, and other mothers’ perspectives on the human papillomavirus vaccine: an Istanbul multicenter study
Source: Front Public Health. 2024 May 2;12:1361509. doi: 10.3389/fpubh.2024.1361509 (PMC11098012; doi:10.3389/fpubh.2024.1361509)
Supplement: Supplementary file 1 [file Data_Sheet_1.docx]

Supplementary Material

Actions Speak Louder than Words; Pediatricians, gynecologists, nurses, and other mothers' perspectives on the human papillomavirus vaccine: an Istanbul multicenter study

Burcu Parlak^1*^, Funda Güngör Uğurlucan^2^, Emine Gülbin Gökçay^1^

^1^Department of Social Pediatrics, Institute of Child Health, Istanbul University, Istanbul, Turkey

^2^Department of Obstetrics and Gynecology, Istanbul Faculty of Medicine, Istanbul University, Istanbul, Turkey

***Correspondence:**Burcu Parlak

burcu.parlak@ogr.iu.edu.tr

**Supplementary file 1 - Questionnaire - Part 1/3- Personal data.**

Dear Participants,

The purpose of this survey study was to compare the knowledge and attitudes of mothers who work as pediatricians and gynecologists with non-physicians regarding the HPV (Human Papilloma Virus) vaccine, as well as the factors that influence these attitudes. Your responses will not be utilized for purposes other than science, and no private information about you will be disclosed. The I.U. Istanbul Faculty of Medicine Clinical Research Ethics Committee file number 2020/1764, the first meeting decision number, and nos. 30 and 1065 serve as the foundation for our investigation.

(survey's introduction)

| 1.Name, surname: .............................................. Your phone number :............................................  2. Your age: ........... (8,14,19)  3. Your marital status: (8,9,20-3)  4. How many children do you have? 1 ( ) 2 ( ) 3 ( ) 4 and above ( ) (8,9,19,23)  5. How old is your child/children (write the age by putting the letter K at the end of the girls and E at the end of the boys)? ........................ (9)  6. Gender of your child/children: Girl ( ) Boy ( ) Both ( ) (9)  7. Your profession? Mom ..................  Father ............... (9)  8. Your title? Specialist/Lecturer () Doctor lecturer () Associate Professor () Professor () (5,10,21)  9. If you are a physician, what is your branch? Gynecology ( ) Pediatrics ( ) (5,7,20,22)  10. Professional experience: 0 – 2 years ( ) , 3-5 years ( ) , 6-10 years ( ) , 11-15 years ( ) , 16 – 20 years ( ) , 21 years and above ( ) (5,7)  11. Are you currently working actively? Yes ( ) No ( ) (19,20)  12. If you are a physician, where do you work? Family Health Center ( ) State Hospital ( ) University/Education Research Hospital () Private University Hospital () Private Practice ( ) Private Clinic () Private Hospital () (7,20,22)  13. Your family's total monthly income: ……………………. (8,10)  14. Smoking: never ( ) , sometimes ( ) , every day ( ) (8,11,20-1)  15. Does anyone close to you have a history of cervical cancer? Yes ( ) No ( ) (11) |
| --- |

**
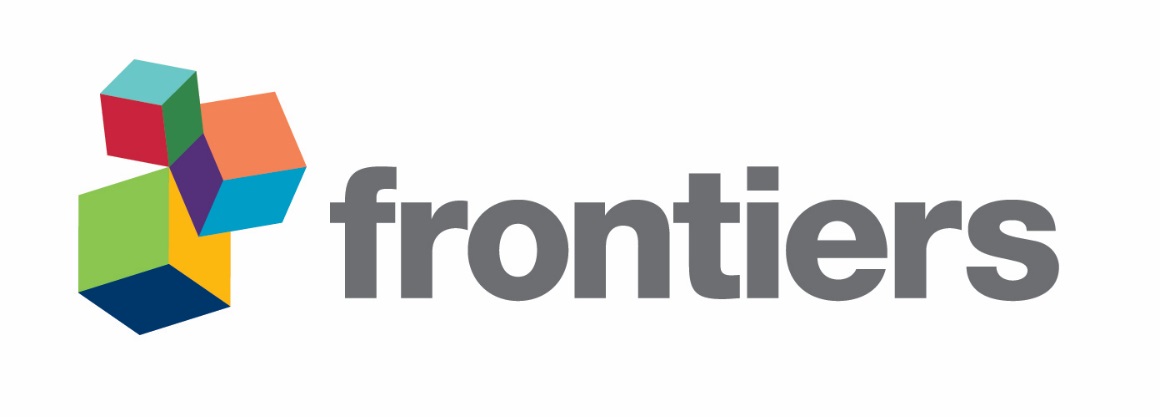
**
