# Supplementary material for: Actions speak louder than words; pediatricians, gynecologists, nurses, and other mothers’ perspectives on the human papillomavirus vaccine: an Istanbul multicenter study
Source: Front Public Health. 2024 May 2;12:1361509. doi: 10.3389/fpubh.2024.1361509 (PMC11098012; doi:10.3389/fpubh.2024.1361509)
Supplement: Supplementary file 2 [file Data_Sheet_2.docx]

Supplementary Material

Actions Speak Louder than Words; Pediatricians, gynecologists, nurses, and other mothers' perspectives on the human papillomavirus vaccine: an Istanbul multicenter study

Burcu Parlak^1*^, Funda Güngör Uğurlucan^2^, Emine Gülbin Gökçay^1^

^1^Department of Social Pediatrics, Institute of Child Health, Istanbul University, Istanbul, Turkey

^2^Department of Obstetrics and Gynecology, Istanbul Faculty of Medicine, Istanbul University, Istanbul, Turkey

***Correspondence:**Burcu Parlak

burcu.parlak@ogr.iu.edu.tr

**Supplementary file 2 - Questionnaire – Part 2/3 – knowledge.**

| 1. From what source did you get your ideas about HPV? (8,19)  In medical school ( )  During specialist training ( )  Medical books, scientific articles and congresses followed after specialization ( )  From other books and publications ( )  From family elders ( )  From my friends ( )  Via social media ( )  2. What is the age of the HPV vaccine target population? Under 9 years old ( ) 9-26 years old ( ) 30 years and above ( ) (5,6,10,14,24)  3. Do you think the price of the HPV vaccine is reasonable? Yes ( ) No ( ) (10,14,20,25)  4. Can HPV be transmitted sexually? Yes ( ) No ( ) (10,13,19,20)  5. Does multiple sexual partners increase the risk of HPV? Yes ( ) No ( ) (10,19)  6. Does using a condom reduce the risk of HPV? Yes ( ) No ( ) (13,14,19)  7. Can HPV cause cervical cancer? Yes ( ) No ( ) (10,13,19,20)  8. Can a person live for years without knowing they have HPV infection? Yes ( ) No ( ) (13)  9. Is HPV rare? Yes ( ) No ( ) (13,25)  10. There are many types of HPV. True ( ) False ( ) (14,20,25)  11. Sexual intercourse at an early age increases the risk of HPV. True ( ) False ( ) (12,25)  12. HPV can be treated with antibiotics/antivirals. True ( ) False ( ) (25)  13. HPV does not cause infection in men. True ( ) False ( ) (12,13,20,25)  14. HPV symptoms are always visible. True ( ) False ( ) (13,20,25)  15. HPV causes genital warts. True ( ) False ( ) (13,14,19,20,25)  16. HPV usually resolves without treatment. True ( ) False ( ) (20,25)  17. Vaccinated girls do not need a smear test when they grow up. True ( ) False ( ) (5,6,8,10,20,25)  18. HPV vaccine protects against many types of cervical cancer. True ( ) False ( ) (14,19,20,25)  19. Someone who is vaccinated against HPV will not get cervical cancer. True ( ) False ( ) (5,6,14,25)  20. HPV can also cause other types of cancer. True ( ) False ( ) (8,10,14,20,23)  21. HPV vaccine protects against genital warts. True ( ) False ( ) (13,19,25)  22. Should only girls be vaccinated? Yes ( ) No ( ) (13,19,20)  23. HPV vaccine is given in 2 doses, 6 months apart, between the ages of 9-14. True ( ) False ( ) (5,14,20)  24. HPV vaccine is given in 3 doses at 0, 2 and 6 months for those aged 15 and over. True ( ) False ( ) (5,14,20)  25. Can you write a few side effects of the HPV vaccine? (8,11,13,14,19,24) |
| --- |

**
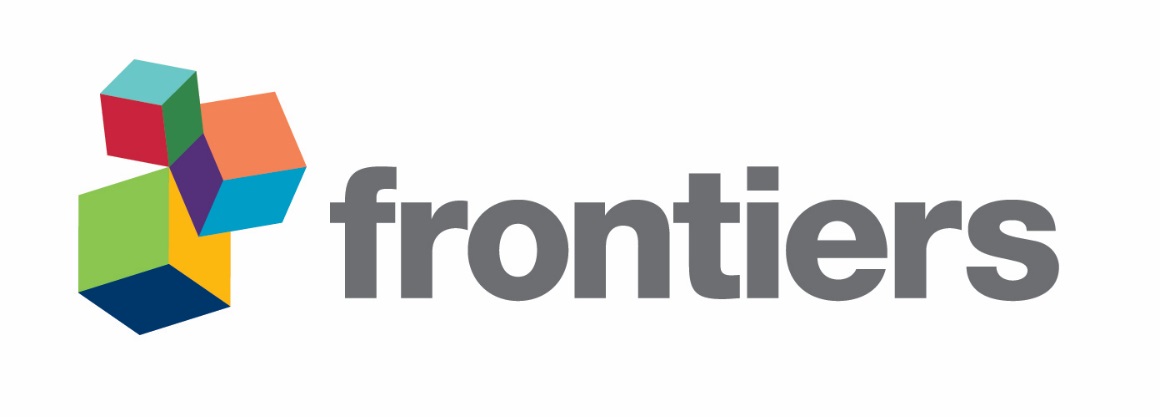
**
