# Supplementary material for: Actions speak louder than words; pediatricians, gynecologists, nurses, and other mothers’ perspectives on the human papillomavirus vaccine: an Istanbul multicenter study
Source: Front Public Health. 2024 May 2;12:1361509. doi: 10.3389/fpubh.2024.1361509 (PMC11098012; doi:10.3389/fpubh.2024.1361509)
Supplement: Supplementary file 3 [file Data_Sheet_3.docx]

Supplementary Material

Actions Speak Louder than Words; Pediatricians, gynecologists, nurses, and other mothers' perspectives on the human papillomavirus vaccine: an Istanbul multicenter study

Burcu Parlak^1*^, Funda Güngör Uğurlucan^2^, Emine Gülbin Gökçay^1^

^1^Department of Social Pediatrics, Institute of Child Health, Istanbul University, Istanbul, Turkey

^2^Department of Obstetrics and Gynecology, Istanbul Faculty of Medicine, Istanbul University, Istanbul, Turkey

***Correspondence:**Burcu Parlak

burcu.parlak@ogr.iu.edu.tr

**Supplementary file 3 - Questionnaire – Part 3/3 – attitude.**

| 1. Have you had the HPV vaccine? Yes ( ) No ( ) (6,8,11,24)  2. Have you vaccinated your child with the HPV vaccine? / Do you want your child to get the HPV vaccine? Yes ( ) No ( ) / Yes ( ) No ( ) (6,8,9,13,19,24)  3. How many of your children have had the HPV vaccine? / How many of your children will you vaccinate against HPV? Girl……. Boy……. / Girl……. Boy…… (9)  4. Which brand of HPV vaccine was made? Cervarix ( ) Gardasil ( ) Gardasil 9 ( ) None ( ) (21,26)  5. Was the HPV vaccine administered at an age-appropriate dose? Yes ( ) No ( ) (11,26)  6. Do you have a smear test regularly? Yes ( ) No ( ) (21,24)  7. If you have not received the HPV vaccine, what is the reason? Side effects ( ) Price ( ) Unnecessary ( ) (11,14,24)  8. Do you recommend the HPV vaccine to your patients or those around you? Yes ( ) No ( ) (6,7,8,14,20,21) |
| --- |

**
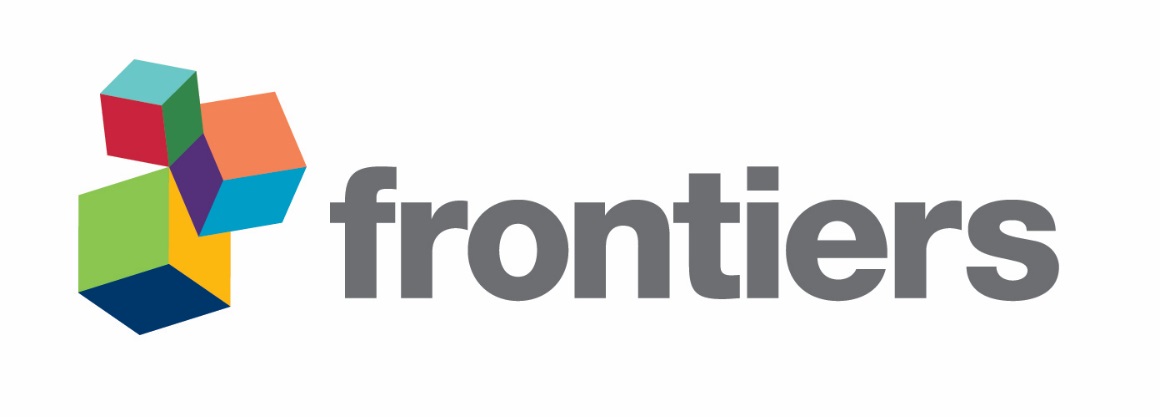
**
