# Supplementary material for: Actions speak louder than words; pediatricians, gynecologists, nurses, and other mothers’ perspectives on the human papillomavirus vaccine: an Istanbul multicenter study
Source: Front Public Health. 2024 May 2;12:1361509. doi: 10.3389/fpubh.2024.1361509 (PMC11098012; doi:10.3389/fpubh.2024.1361509)
Supplement: Supplementary file 4 [file Data_Sheet_4.docx]

Supplementary Material

Actions Speak Louder than Words; Pediatricians, gynecologists, nurses, and other mothers' perspectives on the human papillomavirus vaccine: an Istanbul multicenter study

Burcu Parlak^1*^, Funda Güngör Uğurlucan^2^, Emine Gülbin Gökçay^1^

^1^Department of Social Pediatrics, Institute of Child Health, Istanbul University, Istanbul, Turkey

^2^Department of Obstetrics and Gynecology, Istanbul Faculty of Medicine, Istanbul University, Istanbul, Turkey

***Correspondence:**Burcu Parlak

burcu.parlak@ogr.iu.edu.tr

**Supplementary file 4 - Answers - Part 2/3- Answers about information**

|  | **Group 1**  **(n=71)** | | **Group 2**  **(n=120)** | **Group 3**  **(n=66)** | **N (%)** |  |
| --- | --- | --- | --- | --- | --- | --- |
| **“What is the source of your opinion about HPV?”** |  | |  |  |  | **p=0,0001** |
| **Social media** n (%) | | 0 | **43 (35,8)** | 10 (15,2) | **53 (20,6)** |  |
| **Medicine faculty**  n (%) | | **25 (35,2)** |  | 11(9,2) | 36 (14) |  |
| **Doctor**  n (%) | |  | 13 (10,8) |  | 13 (5,1) |  |
| **Family elders**  n (%) | | 1 (1,4) | 6 (5) | 1(1,5) | 8 (3,1) |  |
| **Friends**  n (%) | | 0 | 9 (7,5) | 2(3) | 11(4,3) |  |
| **During specialist training**  n (%) | | 23 (32,4) | 2(1,7) | 4 (6,1) | 29 (11,3) |  |
| **Books, publications and congresses followed after specialization**  n (%) | | 22 (30,6) | 2 (1,7) | 5(7,6) | 29 (11,3) |  |
| **Other books and publications (TV, newspapers, magazines, non-scientific journals)** | | 0 | 26 (21,7) | **28 (42,4)** | **54 (21)** |  |
| **Not answering** | | 1 (1,4) | 19 (15,8) | 5(7,6) | 25 (9,7) |  |
| **Total** | | 71 | 120 | 66 | 257 |  |
| **What is the age of the target group of the HPV vaccine?** | |  |  |  |  | **p= 0,0001** |
| **Under 9 years old** | | 0 | 5 (4,2) | 3 (4,8) | 8 (3,1) |  |
| **9-26 years old** | | 66 (93) | 54 (45) | 43 (68.3) | 163 (63,4) |  |
| **30 years and over** | | 4 (5,6) | 27 (22,5) | 5 (7,9) | 36 (14) |  |
| **Do not know** | | 1 (1,4) | 34 (28,3) | 12 (19) | 47 (18,3) |  |
| **Not answering** | |  |  | 3 (4,6) | 3 (1,2) |  |
| **Total** | |  |  |  | 257 |  |
| **Is the price of the HPV vaccine reasonable?** | |  |  |  |  | **p=0.002** |
| **No** | | 47 (66,2) | 61 (50,8) | 44 (66,7) | 152 (59,1) |  |
| **Yes** | | 15 (21,1) | 23 (19,2) | 3 (4,6) | 41 (16) |  |
| **Do not know** | | 9 (12,7) | 36 (30) | 10 (15,2) | 55 (21,4) |  |
| **Not answering** | |  |  | 9 | 257 |  |
| **Does multiple sexual partners increase the risk of HPV?** | |  |  |  |  | **p=0.012** |
| **No** | | 0 (0) | 9 (7,5) | 2 (3) | 11 (4,3) |  |
| **Yes** | | 71 (100) | 88 (73,3) | 61 (92,4) | 220 (85,6) |  |
| **Not answering** | | 0 | 23 (19,2) | 3 (4,6) | 26 (10) |  |
| **Is HPV transmitted sexually?** | |  |  |  |  | **p=0.005** |
| **No** | | 0 (0) | 9 (7,5) | 1 (1,5) | 10 (3,9) |  |
| **Yes** | | 71 (100) | 84 (70) | 60 (90,9) | 215 (83,7) |  |
| **Not answering** | | 0 | 27 (22,5) | 5 (7,6) | 32 (12,5) |  |
| **Does using a condom reduce**  **the risk of HPV?** | | | | **p=0.087** | | |
| **No** | | 15 (21) | 23 (19,2) | 6 (9,1) | 44 (17,1) |  |
| **Yes** | | 56 (79) | 74 (61,7) | 55 (83,3) | 185 (72) |  |
| **Not answering** | | 0 | 23 (19,2) | 5 (7,6) | 28 (10,9) |  |
| **Total** | |  |  |  | 257 |  |
| **Can HPV cause cervical cancer?** | |  |  |  |  | **p=0.0001** |
| **No** | | 0 (0) | 14 (11,6) | 2 (3) | 16 (6,2) |  |
| **Yes** | | 71 (100) | 84 (70) | 60 (90,9) | 215 (83,7) |  |
| **Not answering** | | 0 | 22 (18,3) | 4 (6,1) | 26 (10,1) |  |
| **Total** | |  |  |  | 257 |  |
| **Can a person live for years without knowing they have HPV infection?** | |  |  |  |  |  |
| **No** | | 1 (1,4) | 27 (22,5) | 20 (30,3) | 48 (18,7) | **p=0.0001** |
| **Yes** | | 70 (98,6) | 68 (56,7) | 41 (62,1) | 179 (69,7) |  |
| **Not answering** | | 0 | 25 (20,8) | 5 (7,6) | 30 (11,7) |  |
|  | |  |  |  | 257 |  |
| **Is HPV rare?** | |  |  |  |  | **p=0.0001** |
| **No** | | 69 (97,2) | 60 (50) | 50 (75,8) | 179 (69,7) |  |
| **Yes** | | 2 (2,8) | 35 (29,2) | 11 (16,7) | 48 (18,7) |  |
| **Not answering** | | 0 | 25 (20,8) | 5 (7,8) | 30 (11,7) |  |
|  | |  |  |  | 257 |  |
| **There are many types of HPV** | |  |  |  |  |  |
| **No** | | 1 (1,4) | 16 (13,3) | 10 (15,2) | 27 (10,5) | **p=0.003** |
| **Yes** | | 70 (98,6) | 77 (64,2) | 47 (71,2) | 194 (75,5) |  |
| **Not answering** | | 0 | 27 (22,5) | 9 (13,6) | 36 (14) |  |
|  | |  |  |  | 257 |  |
| **Sexual intercourse at an early age increases the risk of HPV** | |  |  |  |  | **p=0.0001** |
| **No** | | 2 (2,8) | 29 (24,2) | 15 (22,7) | 46 (17,9) |  |
| **Yes** | | 69 (97,2) | 62 (51,7) | 46 (69,7) | 177 (68,9) |  |
| **Not answering** | | 0 | 29 (24,2) | 5 (7,8) | 34 (13,3) |  |
|  | |  |  |  | 257 |  |
| **HPV can be treated with**  **antibiotics/antivirals** | | | | **p=0.0001** | | |
| **Yes** | | 8 (11,3) | 42 (35) | 32 (48,5) | 82 (31,9) |  |
| **No** | | 63 (88,7) | 49 (40,8) | 26 (39,4) | 138 (53,7) |  |
| **Not answering** | | 0 | 29 (24,2) | 8 (12,1) | 37 (14,4) |  |
|  | |  |  |  | 257 |  |
| **HPV does not cause infection in men** | |  |  |  |  | **p=0.002** |
| **No** | | 67 (94,4) | 70 (58,3) | 41 (62,1) | 178 (69,3) |  |
| **Yes** | | 4 (5,6) | 21 (17,5) | 16 (24,2) | 41 (16) |  |
| **Not answering** | | 0 | 29 (24,2) | 9 (13,6) | 38 (14,8) |  |
| **HPV symptoms are**  **always visible** | | | | **p=0.005** | | |
| **Yes** | | 0 (0) | 13 (10,8) | 8 (12,1) | 21 (8,2) |  |
| **No** | | 71 (100) | 82 (68,3) | 51 (77,3) | 204 (79,4) |  |
| **Not answering** | | 0 | 25 (20,8) | 7 (10,6) | 32 (12,5) |  |
| **HPV causes genital warts** | |  |  |  |  | **p=0.0001** |
| **Yes** | | 71 (100) | 82 (68,3) | 60 (90,9) | 213 (82,9) |  |
| **No** | | 0 (0) | 12 (10) | 1 (1,5) | 13 (5,1) |  |
| **Not answering** | |  | 26 (21,7) | 5 (7,6) | 31 (12,1) |  |
| **HPV usually resolves without treatment** | |  |  |  |  |  |
| **Yes** | | 27 (38) | 6 (5) | 5 (7,8) | 38 (14,8) | **p=0.0001** |
| **No** | | 44 (62) | 86 (71,2) | 55 (83,3) | 185 (72) |  |
| **Do not know** | | 0 | 1 (0,8) | 1 (1.5) | 2 (0,8) |  |
| **Not answering** | |  | 27 (22,5) | 5 (7,8) | 32 (12,5) |  |
| **Vaccinated girls do not need smear tests when they grow up** | |  |  |  |  | **p=0.045** |
| **Yes** | | 0 (0) | 6 (5) | 2 (3) | 8 (3,1) |  |
| **No** | | 71 (100) | 88 (73,3) | 60 (90,9) | 219 (85,2) |  |
| **Do not know** | | 0 | 1 (0,8) | 1 (1.5) | 2 (0,8) |  |
| **Not answering** | |  | 25 (20,8) | 3 (4,6) | 28 (42,4) |  |
| **HPV vaccine protects against many types of cervical cancer** | |  |  |  |  | **p=0.351** |
| **Yes** | | 61 (86) | 73 (60,8) | 49 (74,2) | 183 (71,2) |  |
| **No** | | 10 (14) | 22 (18,3) | 11 (16,7) | 43 (16,7) |  |
| **Do not know** | | 0 | 1 (0,8) | 1 (1.5) | 2 (0,8) |  |
| **Not answering** | |  | 24 (20) | 5 (7,6) | 29 (11,3) |  |
| **Someone who gets the HPV vaccine will not get cervical cancer** | |  |  |  |  | **p=0.067** |
| **Yes** | | 7 (10) | 19 (15,8) | 15 (22,7) | 41 (16) |  |
| **No** | | 64 (90) | 70 (58,3) | 46 (69,7) | 180 (70) |  |
| **Do not know** | | 0 | 1 (0,8) | 1 (1,5) | 2 (0,8) |  |
| **Not answering** | |  | 30 (25) | 4 (6,1) | 34 (13,2) |  |
| **HPV can also cause other types of cancer** | |  |  |  |  | **p=0.001** |
| **Yes** | | 61 (86) | 55 (45,8) | 43 (65,2) | 159 (61,9) |  |
| **No** | | 10 (14) | 36 (30) | 13 (19,7) | 59 (23) |  |
| **Do not know** | | 0 | 1 (0,8) | 1 (1,5) | 2 (0,8) |  |
| **Not answering** | |  | 28 (23,3) | 9 (13,6) | 37 (14,4) |  |
| **HPV vaccine protects**  **against genital warts** | | | | **p=0.024** | | |
| **Yes** | | 56 (79) | 60 (50) | 52 (78,8) | 168 (65,4) |  |
| **No** | | 13(18.3) | 26 (21,7) | 7 (10,6) | 46 (17,9) |  |
| **Not answering** | | 2 (2.8) | 34 (28,3) | 7 (10,6) | 44 (17) |  |
| **Should only girls be vaccinated?** | |  |  |  |  | **p=0.013** |
| **Yes** | | 7 (9,9) | 21 (17,5) | 18 (27,3) | 45 (17,5) |  |
| **No** | | 64 (90,1) | 79 (65,8) | 41 (62,1) | 184 (71,6) |  |
| **Not answering** | |  | 21 (17,5) | 7 (10,6) | 28 (10,9) |  |
| **HPV vaccine is given in 2 doses, 6 months apart, between the ages of 9-14.** | |  |  |  |  | **p=0.0001** |
| **False** | | 9 (12,7) | 33 (27,5) | 10 (15.2) | 52 (20.2) |  |
| **True** | | 59 (83) | 53 (44,2) | 46 (69,7) | 158 (61.5) |  |
| **Do not know** | | 3 (4,2) | 15 (12,5) | 2 (3) | 20 (7.8) |  |
| **Not answering** | |  | 19 (15,8) | 8 (12) | 27 (10.5) |  |
| **HPV vaccine is given in 3 doses at 0, 2 and 6 months for those aged 15 and over.** | |  |  |  |  | **p=0.011** |
| **False** | | 10 (14) | 31 (25,8) | 15 (22,7) | 56 (21,8) |  |
| **True** | | 57 (80,3) | 52 (43,3) | 38 (57,6) | 147 (57,2) |  |
| **Do not know** | | 4 (5,6) | 11 (9,2) | 2 (3) | 17 (6,6) |  |
| **Not answering** | |  | 26 (21,7) | 11 (16,7) | 37 (14,4) |  |
