# Supplementary material for: Actions speak louder than words; pediatricians, gynecologists, nurses, and other mothers’ perspectives on the human papillomavirus vaccine: an Istanbul multicenter study
Source: Front Public Health. 2024 May 2;12:1361509. doi: 10.3389/fpubh.2024.1361509 (PMC11098012; doi:10.3389/fpubh.2024.1361509)
Supplement: Supplementary file 5 [file Data_Sheet_5.docx]

Supplementary Material

Actions Speak Louder than Words; Pediatricians, gynecologists, nurses, and other mothers' perspectives on the human papillomavirus vaccine: an Istanbul multicenter study

Burcu Parlak^1*^, Funda Güngör Uğurlucan^2^, Emine Gülbin Gökçay^1^

^1^Department of Social Pediatrics, Institute of Child Health, Istanbul University, Istanbul, Turkey

^2^Department of Obstetrics and Gynecology, Istanbul Faculty of Medicine, Istanbul University, Istanbul, Turkey

***Correspondence:**Burcu Parlak

burcu.parlak@ogr.iu.edu.tr

**Supplementary file 4 – References 22-26**

22. Qaqish A, Abdo N, Abbas MM, Saadeh N, Alkhateeb M, Msameh R, et al Awareness and knowledge of physicians and residents on the non-sexual routes of human papilloma virus (HPV) infection and their perspectives on anti-HPV vaccination in Jordan. Plos one, (2023) 18(10), <https://doi.org/10.1371/journal.pone.0291643>

23. Radecki Breitkopf C, Finney Rutten LJ, Findley V, Jacobson DJ, Wilson PM., Albertie M Awareness and knowledge of Human Papillomavirus (HPV), HPV‐related cancers, and HPV vaccines in an uninsured adult clinic population. Cancer Medicine, (2016) 5(11), 3346-3352. https://doi.org/10.1002/cam4.933

24. Siu JYM. Perceptions of and barriers to vaccinating daughters against Human Papillomavirus (HPV) among mothers in Hong Kong. J BMC women's health. 2014;14(1):1-10

25. Liu Z, Zhang L, Yang Y, Meng R, Fang T, Dong Y, Zhan S. Active surveillance of adverse events following human papillomavirus vaccination: feasibility pilot study based on the regional health care information platform in the city of Ningbo, China. Journal of medical Internet research, 2020;22(6).

26. Tatar O, Perez S, Naz A, Shapiro GK, Rosberger Z. Psychosocial correlates of HPV vaccine acceptability in college males: a cross-sectional exploratory study. Papillomavirus Research, 2017; 4, 99-107.

**
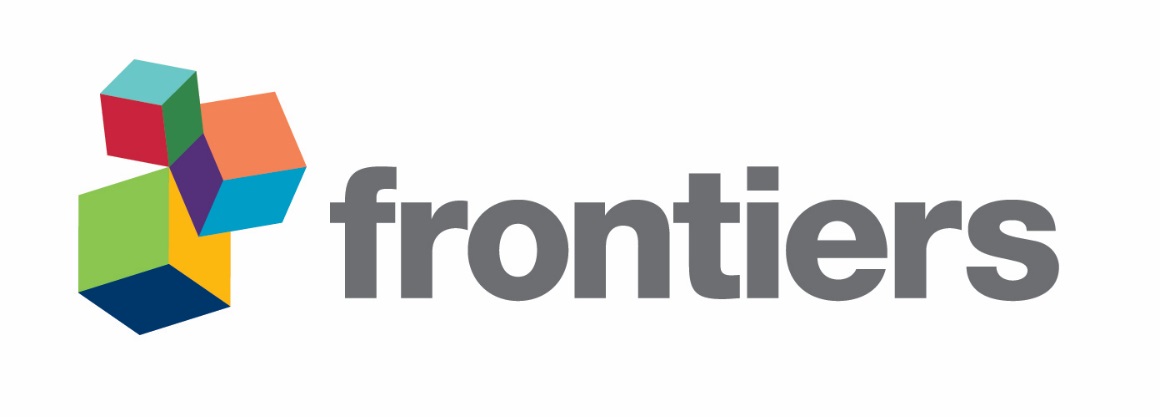
**
